# Concomitant ipsilateral intracapsular and extracapsular femoral neck fracture: a case report

**DOI:** 10.1186/1752-1947-2-68

**Published:** 2008-02-29

**Authors:** Daniel C Perry, Simon J Scott

**Affiliations:** 1 University Hospital Aintree, Longmoor Lane, Fazakerley, Liverpool, UK

## Abstract

**Introduction:**

Intracapsular and extracapsular hip fractures are common amongst elderly patients but simultaneous intracapsular and extracapsular hip fractures are rare.

**Case presentation:**

We present the case of an elderly woman who sustained simultaneous intracapsular and extracapsular hip fractures and describe the complications which ensued following fixation.

**Conclusion:**

Concomitant ipsilateral intracapsular and extracapsular femoral neck fracture is an uncommon injury pattern. It occurs most commonly in osteoporotic patients with low energy falls. Close examination of radiographs must be made to ensure that more subtle fractures are not overlooked and the injury managed appropriately. If doubt exists on initial radiographs further imaging should be considered.

## Introduction

Simultaneous intracapsular and extracapsular hip fractures are rare. There are several previous case reports but in these all fractures had been detected prior to definitive fracture fixation being performed. There has been no comprehensive discussion in the literature collating the details of these cases or suggesting optimal management. These fractures can be difficult to diagnose, and consequently may be incorrectly managed which may result in failure of fixation.

## Case presentation

**Figure 1:**
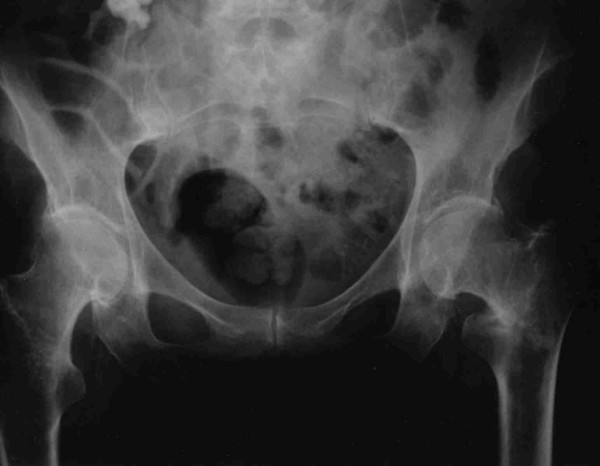
AP radiograph of pelvis.

An 86-year-old woman was admitted following a simple low energy fall in her home. She complained of left hip pain and was unable to weightbear. Examination of her leg revealed a shortened and externally rotated left leg. A plain pelvic radiograph was performed (Figure 1).

A diagnosis of a displaced intertrochanteric fracture was made and the patient was prepared for surgery.

**Figure 2:**
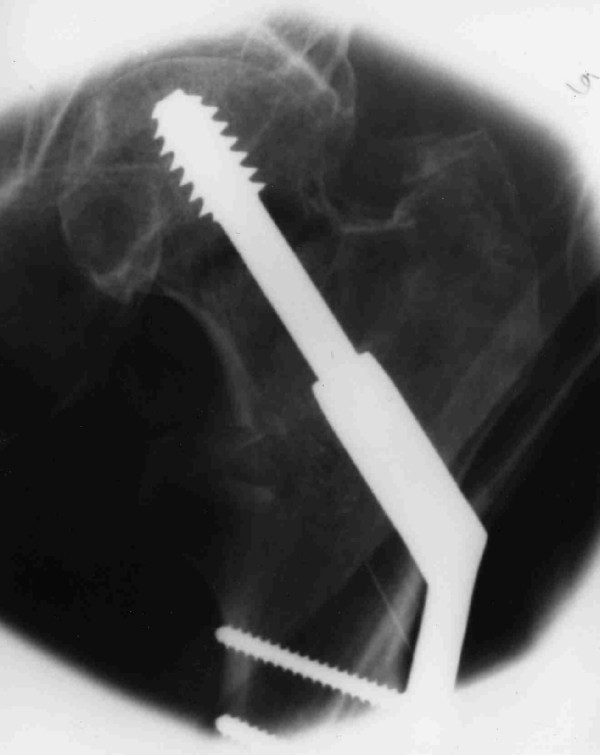
Intraoperative image of the left hip showing the position of DHS components.

Dynamic hip screw (DHS) fixation of the left femur was performed on the next available operating list. Post-operative radiographs confirmed a satisfactory pin position (Figure 2). The tip-apex distance was less than 24 mm.

**Figure 3:**
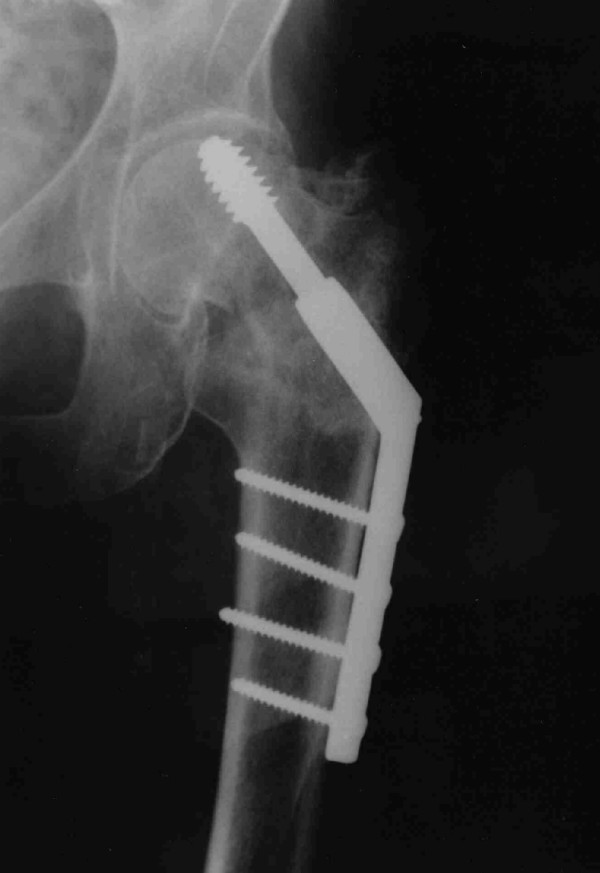
AP Radiograph of left hip depicting failure of fixation and evidence of an intracapsular fracture.

Post-operative mobility was commenced although the patient continued to complain of left hip pain. After ten weeks of rehabilitation and ongoing hip pain a further radiograph was performed (Figure 3).

This radiograph illustrated the presence of a displaced intracapsular femoral neck fracture in addition to the intertrochanteric fracture. The original radiograph (Figure A) was reviewed and it was discovered that this also demonstrated the presence of an undisplaced intracapsular fracture at the time of initial injury but this had not been identified.

Protected weightbearing was commenced but despite this fixation failed at week 12 postoperatively.

Total hip replacement was recommended but the patient refused further surgical intervention.

## Discussion

There are six reports of a simultaneous intracapsular and extracapsular fracture of the hip in the medical literature. More commonly reported is the presence of a subcapital fracture occurring sequentially following DHS fixation, which is thought to arise secondary to poor pin placement leading to a 'stress riser' effect.

Cases of simultaneous intracapsular and extracapsular fracture are thwart with problems relating to diagnosing this injury and debate regarding optimal methods of fixation.

All known cases except one have occurred following low energy falls in elderly osteoporotic patients with the exception of the case of a 54-year-old man who became entangled in an olive press [1] and who sustained this amongst other injuries.

Two of the cases reported in the literature were apparent on presentation on plain radiographs [1,2], two were suspected on initial radiographs and confirmed with further imaging pre-operatively [3,4] and two were identified at the point of surgical intervention by fluoroscopic examination [5,6].

Methods of fixation vary between cases. DHS fixation is the most common means of fixation adopted in five cases, three of which used supplementary anti-rotation devices. One patient underwent fixation with a long stem hemiarthroplasty and Parham bands [6].

All previous studies have reported complete success with fixation although one patient did succumb to an unrelated disease process two months post-operatively [3].

Failure of fixation in the case we describe may be due to the subcapital fracture remaining undiscovered during fixation. In performing the DHS, rotation of the head of the femur may have occurred whilst reaming or inserting the pin. This may consequently have interrupted the blood supply to the head of the femur resulting in an early avascular necrosis and failure of fixation.

## Conclusion

Concomitant ipsilateral intracapsular and extracapsular femoral neck fracture is an uncommon injury pattern. It occurs most commonly in osteoporotic patients with low energy falls. Close examination of radiographs therefore must be made to ensure that more subtle fractures are not overlooked and the injury managed appropriately. If doubt exists on initial radiographs further imaging should be considered. Definitive fracture management should usually be with DHS fixation with either an intraoperative or long-term anti-rotation device in order to minimise the risk of avascular necrosis to the femoral head.

## Abbreviations

DHS – Dynamic Hip Screw

## Competing interests

Both authors confirm that there are no conflicts of interests, including financial and personal relationships with other people, or organisations, that could inappropriately influence (bias) their work.

## Authors' contributions

Both authors were involved in patient management or writing of the manuscript.

## Consent

Written informed consent was obtained from the patient for publication of this case report and accompanying images. A copy of the written consent is available for review by the Editor-in-Chief of this journal
